# Is bilateral radiotherapy necessary for patients with unilateral squamous cell carcinoma of unknown primary of the head and neck region?

**DOI:** 10.1016/j.ctro.2023.100713

**Published:** 2023-12-13

**Authors:** Laura Oebel, Arnulf Mayer, Justus Kaufmann, Daniel Wollschläger, Jan Hagemann, Maximilian Krüger, Heinz Schmidberger

**Affiliations:** a Department of Radiation Oncology, University Medical Center Mainz, Langenbeckstrasse 1, 55131 Mainz, Germany; b Institute of Medical Biostatistics, Epidemiology and Informatics, University Medical Center Mainz, Langenbeckstrasse 1, 55131 Mainz, Germany; c Department of Head and Neck Surgery, University Medical Center Mainz, Langenbeckstrasse 1, 55131 Mainz, Germany; d Department of Oral and Maxillofacial Surgery, University Medical Center Mainz, Langenbeckstrasse 1, 55131 Mainz, Germany

**Keywords:** Head and neck neoplasms, Unknown primary, Radiotherapy

## Abstract

**Background and Purpose:**

Squamous cell carcinoma of unknown primary (SCC-CUP) of the head and neck region remains a clinical challenge, with uncertainty surrounding the necessity of contralateral irradiation of cervical lymphatic drainage in cases of unilateral involvement.

**Materials and Methods:**

A retrospective study was conducted at the Department of Radiation Oncology, University Medical Center Mainz, on a cohort of 50 patients with unilateral SCC-CUP of the head and neck region treated between 2005 and 2019. 30 patients received bilateral and 20 received unilateral cervical radiotherapy. The majority (n = 38, 76 %) were treated with modern IMRT/ VMAT (Intensity-modulated Radiation Therapy/ Volumetric Modulated Arc Therapy) techniques.

**Results:**

After a median follow-up of 64.5 months, locoregional recurrences occurred in 26 % of cases (n = 13/50), all of which were ipsilateral and predominantly within the volume of the previous irradiated CTV (clinical target volume) (85 %, n = 11/13). No patient treated unilaterally developed a contralateral recurrence in the neck. After 3 years, we observed 7 locoregional recurrences in the bilateral irradiated group (n = 7/30, 23 %), and 5 locoregional recurrences in the unilateral irradiated group (n = 5/20, 25 %). After 3 years, 12 patients had died in the bilateral irradiated group (n = 12/30, 40 %), and 7 in the unilateral irradiated group (n = 7/20, 35 %). 7 Patients showed distant metastases after 3 years in the bilateral irradiated group (n = 7/30, 23 %), and 2 in the unilateral irradiated group (n = 2/20, 10 %). Locoregional control (LRC) at 5 years was 66.2 % in the bilaterally irradiated group, and 70.0 % in the unilaterally irradiated group. Overall survival (OS) was 52.6 % (bilateral) and 64.0 % (unilateral). Distant metastasis-free survival (DMFS) was 74.7 % (bilateral) and 84.4 % (unilateral). No significant differences were observed in OS (p = 0.37), LRC (p = 0.91), and DMFS (p = 0.91) between the groups.

Acute toxicity ≥ °2 accordingly CTCAE (Common Terminology Criteria of Adverse Events) was high with 97% while late toxicity ≥ °2 was moderate with 31%. There was no statistically significant difference between the group of unilateral and bilateral irradiated patients.

**Conclusion:**

These data suggest that contralateral cervical irradiation may be of limited benefit in patients with SCC-CUP, as recurrences occured ipsilaterally, and predominantly within the area of prior irradiation. Unilateral irradiation seems to be adequate for carefully selected patients.

## Highlights

• The risk of contralateral recurrence in unilateral cervical SCC-CUP is low.
• Recurrences occur primarily in the area of previous irradiation.
• We found no **statistical significant difference in OS, LRC, and DMFS between** bilateral and unilateral cervical radiotherapy.
• Acute toxicity ≥ °2 was severe with 97% while late toxicity ≥ °2 was moderate with 31% of cases.

## Introduction

Cancer of unknown primary (CUP) is a challenging clinical scenario, accounting for 3–5 % of all malignancies, characterized by the presence of metastases without identification of a primary tumor [1]. In 31 % of the cases, CUP is localized in the head and neck region predominantly as SCC (65–76 %) and corresponds to 2–3 % of all head and neck tumors [2], [3], [4]. There is no uniform therapy standard with regard to the extent of the radiation volumes [5], [6], [7], [8], [9], [10] in the case of unilateral cervical manifestations. These unilateral cervical manifestations are the majority of cases with 75–90 % of all SCC-CUPs [5], [11]. Historically, bilateral neck irradiation for SCC-CUP has been considered standard of care [12]. In order to minimize treatment-related toxicity, efforts have been made to determine whether unilateral irradiation provides the same oncologic outcome. A prospective randomized trial between both options started recruiting in 2002 but failed to show any results due to a lack of patient enrollment [13]. Nevertheless, there is growing evidence in head and neck cancer, that bilateral irradiation of the elective neck might be an overtreatment. A *meta*-analysis from Al-Mamgani et al. including 1116 patients with oropharynx carcinoma showed that contralateral recurrences in unilaterally irradiated patients occurred in 2.4 % only [14]. Recently, a prospective study has shown that SPECT-CT-guided unilateral irradiation of the elective lymphatic drainage pathways of head and neck cancer results in only one contralateral recurrence of 41 unilateral treated patients [15]. With regard to CUP, de Ridder et al. demonstrated excellent regional control rates of 90 % in 80 patients treated with IMRT, including 11 patients irradiated to the unilateral neck only resulting in no contralateral failure [16].

With the present analysis, we publish the data from our department, which provides an indication of the necessary extent of irradiation in the context of the already published literature on CUP.

## Materials and Methods

### Patient characteristics

We analyzed all SCC-CUP patients with unilateral cervical metastases of the head and neck region who received radiotherapy with or without simultaneous chemotherapy at our institution between 03/2005 and 06/2019. This retrospective analysis aimed at evaluating the outcome of bilateral vs. ipsilateral radiotherapy to the cervical lymphatic drainage. The following endpoints were analyzed: OS, LRC, DMFS and metachronous occurrence of a primary mucosal tumor.

Inclusion criteria were histology of SCC, unilateral cervical metastases, and absence of metastatic disease. We excluded patients with a previous history of radiotherapy, and those referred for palliative radiotherapy. All but two patients received panendoscopy. All analyzed CUPs were re-staged according to the 8th edition of the TNM classification without adjustment to the p16 status [17]. Thirty patients were treated with bilateral neck radiotherapy (n = 30/50, 60 %), and twenty patients received unilateral radiotherapy (n = 20/50, 40 %). All but one patient with bilateral cervical irradiation received bilateral irradiation of the pharyngeal mucosa. The patients with unilaterally irradiated neck were divided as follows: 60 % (n = 11/20) received unilateral mucosal radiotherapy, 40 % received bilateral mucosal radiotherapy (n = 8/20), and one patient received no mucosal radiotherapy (Table 1).

**Table 1:** Patient characteristics. ECOG = Eastern Cooperative Oncology Group, HPV = human papilloma virus, ECE = extracapsular extension.

**Patient characteristics**			
	**Bilateral** (n = 30)	**Unilateral** (n = 20)	**Test Statistic**
**Median age**	64.8	66.2	p = 0.47
**ECOG**			p = 0.02
0	15/30 (50 %)	7/20 (35 %)	
1	15/30 (50 %)	8/20 (40 %)	
2	0	5/20 (25 %)	
**Adjuvant**	25/30 (83 %)	14/20 (70 %)	p = 0.26
**Definitive**	5/30 (17 %)	6/20 (30 %)	p = 0.26
**N-Stage**			p = 0.38
1	8/30 (27 %)	7/20 (35 %)	
2a	3/30 (10 %)	2/20 (10 %)	
2b	5/30 (17 %)	6/20 (30 %)	
3a	4/30 (13 %)	0	
3b	10/30 (3**3** %)	5/20 (25 %)	
**Chemotherapy**			p = 0.01
None	5/30 (17 %)	14/20 (70 %)	
Cisplatin	18/30 (60 %)	5/20 (25 %)	
Cisplatin + 5-FU	4/30 (13 %)	0	
Cetuximab	3/30 (10 %)	1/20 (5 %)	
**PET-CT available**	17/30 (57 %)	14/20 (70 %)	p = 0.34
**Neck dissection**			p = 0.39
Selective	8/30 (27 %)	9/20 (45 %)	
Modified/ radical	18/30 (60 %)	8/20 (40 %)	
Biopsy	4/30 (13 %)	3/20 (15 %)	
**Neck dissection side**			p = 0.53
Unilateral	23/30 (77 %)	13/20 (65 %)	
Bilateral	3/30 (10 %)	4/20 (20 %)	
None	4/30 (13 %)	3/30 (10 %)	
**Number of tumor-involved lymph node levels**			p = 0.11
1	12/30 (40 %)	11/20 (55 %)	
2	10/30 (33 %)	6/20 (30 %)	
3	3/30 (10 %)	3/20 (15 %)	
4	2/30 (7 %)	0	
Undefined	3/30 (10 %)	0	
**Tumor-involved lymph node levels**			p = 0.429
Level Ib	8/53 (15 %)	5/32 (16 %)	
Level II	22/53 (42 %)	13/32 (41 %)	
Level III	12/53 (23 %)	8/32 (25 %)	
Level IV	5/53 (9 %)	2/32 (6 %)	
Level V	3/53 (6 %)	1/32 (3 %)	
Level VIII	2/53 (4 %)	2/32 (6 %)	
Level X	0	1/32 (3 %)	
Undefined	1/53 (2 %)	0	
**Radiotherapy mucosa**			p **<** 0.01
None	0	1/20 (5 %)	
Unilateral	1/30 (3 %)	11/20 (55 %)	
Bilateral	29/30 (97 %)	8/20 (40 %)	
**HPV**			p = 0.89
Negative	9/30 (30 %)	5/20 (25 %)	
Positive	6/30 (20 %)	5/20 (25 %)	
Not determined	15/30 (50 %)	10/20 (50 %)	
**ECE**	12/30 (40 %)	6/20 (30 %)	p = 0.47
**Smoking**			p = 0.44
No	11/30 (37 %)	11/20 (55 %)	
Yes	15/30 (50 %)	7/20 (35 %)	
Unknown	4/30 (13 %)	2/20 (10 %)	
**Alcohol**			p = 0.32
None/ social	22/30 (73 %)	11/20 (55 %)	
Yes	4/30 (13 %)	6/20 (30 %)	
Unknown	4/30 (13 %)	3/20 (15 %)	

30 % of the patients (n = 15/50) received a complete mucosal irradiation of nasopharynx, oropharynx, and hypopharynx. These patients had been treated in the first half of the examination period (2005–2012), except for the last patient with total mucosal irradiation that was treated in 2014. Afterwards, the location of the potential primary tumor was deducted from the tumor-involved LK levels according to the anatomical studies of the lymphatic drainage of the head and neck, published by Grégoire et al. [18], and the radiation target war limited to the corresponding subsite of the mucosa. 24 % of the patients (n = 12/50) received irradiation to two subsites of the mucosa, and 44 % (n = 22/50) received irradiation to one subsite of the mucosa only. The most frequently irradiated mucosal subsite was the oropharynx in 47 of 49 cases. In 20 patients, only the oropharyngeal mucosa was irradiated.

The decision of unilateral versus bilateral cervical irradiation and radiation of the subsite of the mucosa was based at the discretion of the attending radiation oncologist.

A regular follow-up examination every 3 months for the first 2 years after irradiation and every 6 months for up to 5 years was provided. Regular clinical examinations as well as ultrasound examinations and CT or MRI scans of the neck were conducted by the clinićs own otorhinolaryngology and were taken up by most patients. Systematic follow-up examinations were also offered by our Department of Radiation Oncology in order to document and treat any side effects.

### Statistical analysis

All survival endpoints were defined as time spans starting at initial diagnosis using Kaplan-Meier plots. OS was defined as lasting until death or last contact, LRC (included metachronous mucosal primary tumors as well as lymphogenic recurrence) was defined as lasting until cervical recurrence or last contact, and DMFS until the occurrence of metastases, death, or last contact. Unilateral versus bilateral radiotherapy was tested for statistical significance in univariate Cox regression analyses with respect to OS, LRC, and DMFS. The Wilcoxon Mann-Whitney *U* test was used for comparing continuous outcomes between groups. Group comparisons for categorical variables were carried out using chi-squared tests. P-values less than 0.05 were considered statistically significant, without adjustment for multiple testing. Tests were two-sided. In the toxicity analysis, unpaired t-tests were performed to determine differences between the two irradiation groups. Results should be regarded as exploratory. Data analysis was carried out using the statistical environment jamovi, version 2.3 [19].

### Treatment planning

Clinical target volumes were defined as follows: CTV1 corresponded to the region with macroscopic tumor, or regions of extracapsular extension (ECE) or R1/2-resection. CTV2 was outlined as the whole lymph node level of the lymph node metastases. CTV3 contained the suspected mucosal primary tumor as described in *patient characteristics* and the low-risk cervical lymphatic drainage according to Grégoire et al. [20], [21], [22].

CTV1 was treated with 60–64 Gy in the case of adjuvant radiotherapy and with 70 Gy in the setting of macroscopic tumor. Treatment was applied in 2.0 Gy fractions to the CTV1 with a median total dose of 64 Gy in the adjuvant situation. CTV2 dose was 60–64 Gy in 1.8–2.13 Gy, and the median total equivalent dose to 2.0 Gy fractions (EQD2) was again 64 Gy. For CTV3, the prescribed dose ranged between 50 Gy and 56 Gy, fractionated in 1.6–2 Gy, with a median EQD2 of 53.1 Gy. In case of ECE, R1/2-resection status, and in the primary situation, concomitant platinum-based chemotherapy was applied [23]. Patients with contraindications to platinum-containing chemotherapy received Cetuximab [24], [25].

**Fig. 1:**
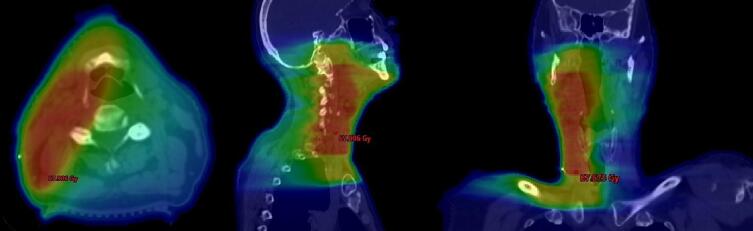
Dose distribution of a VMAT-plan of an 56-year-old patient with T0 N2b (8/30) M0 SCC-CUP in axial, sagittal, and coronal plane. After selective neck dissection of the right side, the patient was treated to the unilateral mucosa and the unilateral elective lymphatic drainage with a two-step dose concept up to 64 Gy (54 Gy in 25 fractions, additional boost volume of 10 Gy in 5 fractions). The patient is recurrence-free 11 years after the end of radiotherapy.

Fig. 1 shows a dose distribution of a VMAT-plan of an 56-year-old patient with T0 N2b M0 SCC-CUP that was treated to the unilateral neck.

## Results

**Fig. 2:**
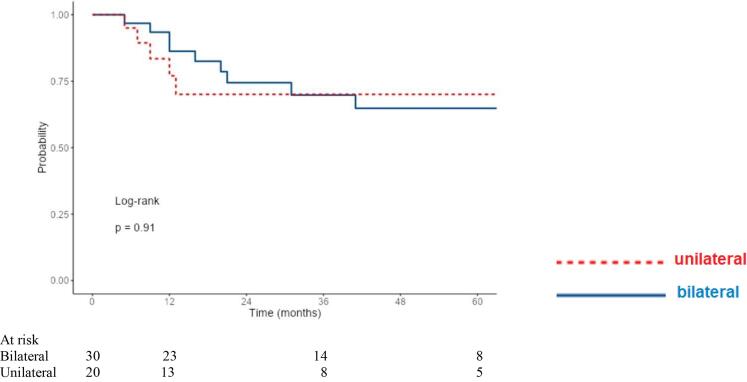

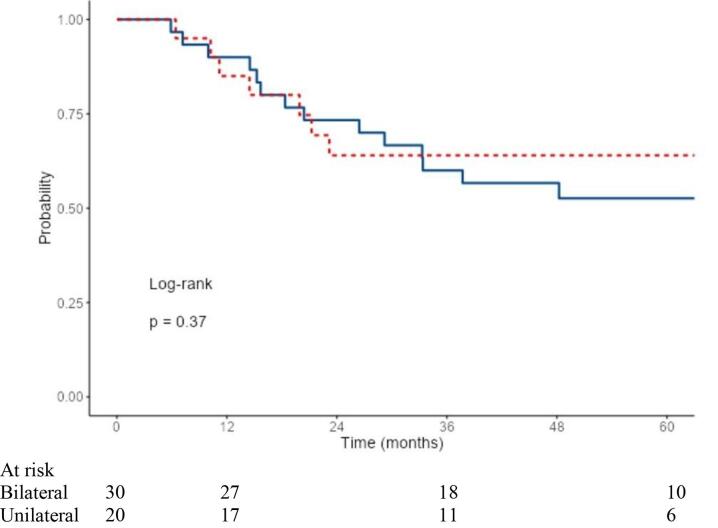

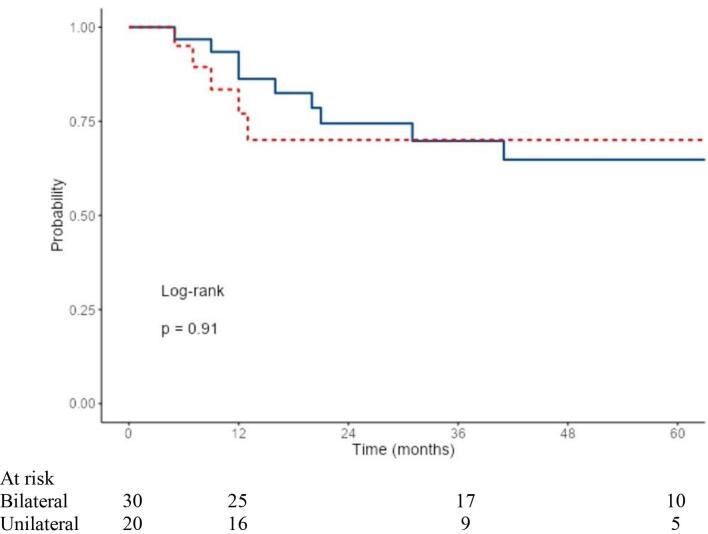
a. LRC in the unilateral vs. bilateral irradiated group. b. OS in the unilateral vs. bilateral irradiated group. c. DMFS in the unilateral vs. bilateral irradiated group.

The median estimated follow-up time was 64.5 months by the reverse Kaplan Meier method. Median time from end of radiotherapy to death was 20 months. No systematic differences in tumor stages were observed between the unilateral and bilateral irradiation groups. However, it was found that the unilaterally irradiated group appeared clinically in a reduced general condition (Table 1). Despite worse prognostic initial conditions with significantly reduced ECOG as well as a reduced radiotherapy volume in terms of the extent of mucosal and cervical radiotherapy, and significantly fewer cases of combined radiochemotherapy (see details in Table 1), OS (p = 0.37), LRC (p = 0.91), and DMFS (p = 0.91) were not worse in the unilaterally irradiated group (Figs. 2a–c).

3-year OS was 60.0 % and 52.6 % after 5 years (bilateral), and 64.0 % after 3 and 5 years each (unilateral) in the groups (Fig. 2b). LRC at 3 and 5 years was 71.7 % and 66.2 % in the bilaterally irradiated group, and 70.0 % and 70.0 % in the unilaterally irradiated group (Fig. 2a). In total, locoregional relapse occurred in 26 % of the patients (n = 13/50). DMFS after 3 and 5 years each was 74.7 % (bilateral) and 84.4 % (unilateral) (Fig. 2c).

All locoregional recurrences occurred ipsilaterally (n = 13/50, 26 %), and predominantly in the CTV (clinical target volume) of the previous irradiation (n = 11/13, 85 %), and were histologically confirmed. Another recurrence occurred directly at the field edge of the PTV (planning target volume) (n = 1/15, 7 %), and one occurred outside the radiation target volume (n = 1/15, 7 %).

Eight recurrences were observed in the bilaterally irradiated group (n = 8/30, 27 %), and five recurrences in patients treated unilaterally (n = 5/20, 25 %). No patient treated unilaterally developed a contralateral recurrence in the neck. Distant metastases (n = 9/50, 18 % of cases) occurred more frequently in the bilaterally irradiated group (n = 7/30, 23 % of cases) than in the unilaterally irradiated group (n = 2/20, 10 % of cases). Similar to the cervical recurrences, metachronous mucosal primary tumors (n = 3/50, 6 %) were located within the irradiated ipsilateral mucosa (one case each located in the buccal mucosa, at the base of the tongue, and at the base of the tongue and the ipsilateral tonsil lodge simultaneously). All metachronous mucosal primary tumors occurred in the group of bilateral cervical radiotherapy. Two of these patients with metachronous mucosal primary tumor were initially treated by VMAT (n = 2/3, 66 %), and one with 3D-technique (n = 1/3, 33 %).

### Toxicity

Toxicity data were analyzed separately for the unilateral vs. bilateral cervical radiation group. Patients receiving unilateral radiotherapy showed partially less side effects, although no significant difference could be found. See details in Table 2.

**Table 2:** Acute and late toxicity after radiotherapy of CUP.

**Acute Toxicity during and within the first three months after radiotherapy**			
	unilateral (n = 20)	bilateral (n = 30)	Test Statistic
	Grade ≥ 2	Grade ≥ 2	
Mucositis	14 (70 %)	22 (73 %)	p = 0.80
Oral pain	11 (55 %)	16 (53 %)	p = 0.91
Xerostomia	5 (25 %)	9 (30 %)	p = 0.71
Dysgeusia	6 (30 %)	12 (60 %)	p = 0.48
Nausea	1 (5 %)	6 (20 %)	p = 0.14
Radiodermatitis	7 (35 %)	7 (23 %)	p = 0.38
Dysphagia	14 (70 %)	25 (83 %)	p = 0.27
Overall Grade ≥ 2 Toxicity	19 (95 %)	29 (97 %)	p = 0.77
**Late toxicity later than three months after radiotherapy was recorded for 36 of 50 patients**			
	unilateral (n = 15)	bilateral (n = 21)	Test Statistic
	Grade ≥ 2	Grade ≥ 2	
Oral pain	1 (7 %)	0	p = 0.24
Xerostomia	3 (20 %)	2 (10 %)	p = 0.39
Dysgeusia	3 (20 %)	3 (14 %)	p = 0.66
Nausea	1 (7 %)	0	p = 0.24
Dysphagia	1 (7 %)	1 (5 %)	p = 0.81
Lymphedema	1 (7 %)	4 (19 %)	p = 0.49
Fibrosis	0	1 (5 %)	p = 0.41
Osteoradionecrosis	0	1 (5 %)	p = 0.41
Esophageal stenosis with bougienage	0	1 (5 %)	p = 0.41
Overall Grade ≥ 2 Toxicity	4 (27 %)	7 (34 %)	p = 0.68

## Discussion

The present study including only patients with CUP of SCC histology and only unilateral cervical lymph node metastases compares favorably with other studies that included multiple histologies and patients with bilateral cervical lymph nodes. This applies in particular to the analysis of uni- vs bilateral radiotherapy and especially with regard to the possibility of reducing the irradiated volume. Most of the previously published cohorts with CUP have been irradiated with conventional techniques. We present the results of a large unicentric patient collective with cervical SCC-CUP irradiatied mostly with modern radiotherapy techniques.

No significant difference in OS was observed between bilaterally and unilaterally irradiated patients. This result is consistent with data from the literature where no OS benefit was shown for either group [8], [9], [10], [26], [27], [28], [29]. We furthermore found no difference in local control between unilaterally and bilaterally irradiated patients, consistent with recent studies [5], [8], [9], [27], [30]. Two older studies showed a benefit in favor of bilateral irradiation in a patient population treated with 2D/3D irradiation techniques [6], [26]. Grau et al. and many of the individual analyses used by Liu et al. for their *meta*-analysis, compared unilateral radiotherapy of the involved neck without any mucosa irradiation to bilateral radiotherapy including radiotherapy of the mucosa. Thus, not only a lymph node recurrence but also the emerge of a metachronous primary tumor will worsen the local control of the unilaterally irradiated group and may explain the worse local control in the unilaterally irradiated group.

We found that local recurrences occurred in all cases on the initially affected side of the neck, and in 85 % in the former CTV. This is comparable to other studies showing none [31], [32], [33], [34], [35] or only a single [10], [27], [36] contralateral recurrence among the unilaterally irradiated patients. Some older cohorts form the 2D era describe higher rates of contralateral recurrences from 10 % up to 15.6 % [8], [26], [37]. In addition to technical advances in radiation modalities, technical advances in diagnostic imaging (e.g., PET/CT) should be noted. Thus, it is possible that a “contralateral recurrence” already present at diagnosis tended to stay undetected at earlier times, leading to an undertreatment with unilateral radiotherapy. The observation that lymphatic recurrences occur almost exclusively in sites that received high radiotherapy doses is also evident in various other studies [8], [10], [28], [38], [39].

The occurrence of emerging primaries can be decreased with radiotherapy to the mucosa of the putative primary tumor region [5], [6], [40]. Three emerging primaries (6 %) were observed in our study within 5 years post-radiotherapy, comparably with other series describing rates between 0 % and 21 % after mucosal radiotherapy [8], [13], [16], [30], [41]. Analogous to the cervical lymph node recurrences, all primary tumors detected in our study occurred on the side of initial tumor involvement. Our data support the thesis of Harper et al. that ipsilateral radiotherapy of the mucosa is sufficient. In their analysis, mucosal site failures were located in the midline or on the same side as the initially positive nodes, if only one side of the neck was clinically positive at presentation, in seven of eight patients [40]. Among 304 patients, Pflumio et al. showed no statistical difference between pan-mucosal and selective mucosal irradiation [5].

Due to the intersecting lymphatic drainage area in the head and neck region, bilateral radiotherapy of the elective lymphatic drainage has been the standard of care since the 1960 s [42]. Nonetheless, several analyses with head and neck carcinoma treated to the ipsilateral neck only show that the incidence of contralateral failure is low [15], [43], [44] of which the largest a *meta*-analysis of 1116 patients with a contralateral failure of only 2,4%, even including T3-tumors [42], supporting evidence that contralateral neck radiotherapy can safely be omitted in selected cases.

Regarding CUPs, for patients with unilateral involvement of a single node and no evidence of ECE, unilateral cervical radiotherapy may be considered according to the newest ASCO Guidelines [12]. Biau et al. also recommend unilateral radiotherapy for selected patients with low tumor burden (N1/ N2a) [45]. Based on our data we recommend unilateral radiotherapy for most patients with unilateral SCC-CUP after an individual risk analysis.

Concerning toxicity, it seems compelling that a larger irradiation target volume in the sense of bilateral irradiation causes more side effects than unilateral irradiation. Nevertheless, this is not reflected statistically in our data. First, the small number of patients makes it difficult to detect statistical effects. In addition to the severity of the side effects, the volumetric extent of the radiodermatitis, which we were not able to collect retrospectively, would also have been of interest. Complicating matters, the documentation of side effects in a retrospective analysis is based on a certain degree of imprecision.

## Limitations

In addition to its retrospective design, a major limitation is the relatively small number of patients in a recruiting period of 14 years due to the rarity of the disease. The heterogeneity of the patient population must be taken into account. The entity CUP itself is based on a certain heterogeneity, as it comprises tumors at different locations in the head and neck area with primary tumors of different radiosensitivity. The p16- status, that has only recently been consistently collected, should be emphasized here. Although there is limited statement possible, the number of HPV-associated tumors in our collective although seems lower compared to recent American collectives of head and neck tumors [46], which may also explain the relatively high number of local recurrences. Furthermore, adjuvant and definitive radiotherapy patients with correspondingly different dose concepts were included in the analysis. Only 62 % of patients received PET-CT staging, and no systematic association with tumor stage was evident in this regard.

## Conclusion

SCC-CUP presents in a majority of cases with unilateral cervical involvement at diagnosis. During the further course of the disease, tumor recurrences continued to occur ipsilaterally in our study.

We analyzed unilateral vs. bilateral irradiated unilateral SCC-CUP patients and found no significant differences in OS, LRC and DMFS. No patient treated unilaterally developed a contralateral recurrence in the neck. Considering the limitations of the study, our data support the recommendation of unilateral radiotherapy for carefully selected CUP patients. We showed high rates of acute toxicity with 97 % ≥ °2 accordingly CTCAE while late toxicity ≥ °2 was moderate with 31 %. Although a reduced toxicity must be assumed for unilateral vs. bilateral irradiation, this was not statistically proven in our collective.

The recurrence analysis of our patients is suggesting that further improvements of treatment for CUP in the head and neck region should be aiming at intensifying multimodal systemic therapy to overcome radioresistance. Combined radioimmunotherapy could represent a decisive development in this regard.

**Declarations**.

The final protocol was approved by the independent Ethics Commission of the State Chamber of Medicine in Rheinland-Pfalz (MZ-2020–15219).

Consent for publication: Not applicable.

Availability of data and material: All data generated or analyzed during the current study are included in this article.

## Funding

None.

## Declaration of competing interest

The authors declare that they have no known competing financial interests or personal relationships that could have appeared to influence the work reported in this paper.
